# Ground beetle fauna of flower strips and forest edges in northern German lowlands’ conventional agricultural landscapes (Coleoptera, Carabidae)

**DOI:** 10.3897/BDJ.13.e161282

**Published:** 2025-08-26

**Authors:** Swantje Grabener, Estève Boutaud, Claudia Drees, Stephan Gürlich, Werner Härdtle, Lena Husemann, Martin Kubiak, Christin Juno Laschke, Sina Remmers, Pascale Zumstein, Thorsten Assmann

**Affiliations:** 1 Institute of Ecology, Leuphana University, Universitätsallee 1, 21335 Lüneburg, Germany Institute of Ecology, Leuphana University, Universitätsallee 1 21335 Lüneburg Germany; 2 Ecologie et Dynamique des Systèmes Anthropisés (EDYSAN), Université de Picardie Jules Verne, 80037 Amiens, France Ecologie et Dynamique des Systèmes Anthropisés (EDYSAN), Université de Picardie Jules Verne 80037 Amiens France; 3 University of Sussex, School of Life Sciences, Brighton, BN1 9RH, United Kingdom University of Sussex, School of Life Sciences Brighton, BN1 9RH United Kingdom; 4 Verein für Naturwissenschaftliche Heimatforschung zu Hamburg e.V., Martin-Luther-King-Platz 3, 20146 Hamburg, Germany Verein für Naturwissenschaftliche Heimatforschung zu Hamburg e.V., Martin-Luther-King-Platz 3 20146 Hamburg Germany; 5 Landesbetrieb für Wasserwirtschaft und Hochwasserschutz Sachsen-Anhalt (LHW), Otto-von-Guericke-Straße 5, 39104 Magdeburg, Germany Landesbetrieb für Wasserwirtschaft und Hochwasserschutz Sachsen-Anhalt (LHW), Otto-von-Guericke-Straße 5 39104 Magdeburg Germany; 6 Institute of Cell- and Systems Biology of Animals, University of Hamburg, Martin-Luther-King-Platz 3, 20146 Hamburg, Germany Institute of Cell- and Systems Biology of Animals, University of Hamburg, Martin-Luther-King-Platz 3 20146 Hamburg Germany

**Keywords:** agriculture, datasets, Barber, pitfall traps, corn fields, corridors, pest management, ecosystem services

## Abstract

**Background:**

Ground beetles are present in most terrestrial ecosystems and fulfil key functions, especially as many species are important predators, contributing to natural pest control in agricultural landscapes. However, intensive agriculture, which combines monocultures and synthetic inputs, has been shown to have negative effects on insect diversity and abundance. To counteract insect decline, numerous measures are being implemented and tested at national scales. These also include flower strips which might have the potential to provide suitable habitats and connect beneficial insects’ populations across agricultural landscapes. Especially if flower strips are located along forest edges, they could reinforce synergy functions and, by that, reduce the barrier effect of conventional agricultural fields. Within the framework of a two-year project, ground beetles were assessed in corn fields [*Zea mays*, Linnaeus 1753], grown for biogas production, with or without a two-seed mixture flower strip as well as in the adjacent forest. Study sites were situated within conventional fields typical for the agricultural landscapes in northern German lowlands in direct proximity to the Nature Reserve Lüneburger Heide.

**New information:**

We provide data on 34,413 specimens belonging to 93 ground beetle species. None of these species is evaluated in the IUCN Red List at the European level, but four species have been classified as being *Near Threatened* within Germany. At the level of the Federal State Lower Saxony, four species are classified as *Endangered*, nine species as *Vulnerable* and one as *Near Threatened*, highlighting the importance of this dataset also for conservation purposes.

This dataset contributes to the knowledge of Central European carabid diversity and distribution, especially within agricultural landscapes. It supports the development of national and European Red Lists. Despite all sites being placed within conventional corn fields, the study area is in direct proximity to the Nature Reserve Lüneburger Heide where threatened heath landscapes exhibit rare carabid species, which could possibly benefit from adapted agricultural management strategies at its borders or even disperse via suitable corridors provided.

## Introduction

The number of studies underpinning terrestrial insect decline is increasing although trends are not unidirectional for taxonomic and functional entities (van Klink et al. 2020, Staab et al. 2023). Amongst others, the high-profile study on the decline of insects by Hallmann et al. (2017), which had a major impact on the general public and raised awareness for insect decline, led to consequences in politics and provided the impetus to promote various targeted greening measures to improve the living conditions of insects. Land-use changes within the last decades are considered one of the most important drivers of insect decline, for example, due to the adverse effects of large-scale monocultures with high amounts of synthetic inputs (Tscharntke et al. 2012, Fiser et al. 2025). Declining biodiversity in agricultural landscapes poses not only a problem for natural communities themselves, but also has negative consequences for agriculture (Geiger et al. 2010) – one of which is that natural pest controllers are declining. Ground beetles (Coleoptera, Carabidae) are important predators that shape arthropod communities by top-down processes and can significantly reduce pest populations in crops (Collins et al. 2002, Symondson et al. 2002, Albrecht et al. 2020, Serée et al. 2021).

Conservation strategies involve marginal strips at field edges, such as flower strips, where synthetic inputs are not applied and no or just an extensive management is carried out. Those could provide a temporal feeding or even source population habitat for ground beetles (Rischen et al. 2023). However, the effectiveness of these strips depends on several factors, such as its location and arrangement within the agricultural landscape matrix, as well as flower strip management characteristics which involve, for example, seed mixture composition (Cole et al. 2008, Haaland et al. 2011, Schütz et al. 2022). In addition to flower strips providing refuge and overwintering habitat for several open landscape species (MacLeod et al. 2004), they may support several ecosystem functions, such as natural pest control or pollination by acting as ecotones in the agricultural landscape (Fournier and Loreau 2001, Baum et al. 2004, Pedley and Dolman 2020). This is particularly important for species that have a low dispersal capacity, for example, brachypterous beetles, many of which are forest specialists (Duflot et al. 2014). These are known to migrate temporarily from forests to open land habitats, whereas open landscape species rarely move into forests (Lacasella et al. 2014, Magura 2017). Due to this spillover, the adjacent habitat also influences the ground beetle community of the habitat under consideration (Schneider et al. 2016). This effect decreases with distance from the habitat boundaries, which means that especially large-scale monocultures can act as a barrier for some species within the agricultural matrix (Boetzl et al. 2024). Furthermore, forest edges inherit specific carabid assemblages which are neither found in forests nor in the crops and, by that, contributing to overall γ-diversity at the landscape level (Magura et al. 2001, Leslie et al. 2014).

In the present project, we investigated the ground beetle assemblage in two types of flower strips sown with different seed mixtures in comparison to corn fields, both directly adjacent to forest edges. The ground beetle assemblages were also recorded in the adjacent forest interiors in order to detect possible synergy effects in both directions. The objective of the present paper is to publish the raw data of carabid specimens sampled throughout the project. This dataset serves as a resource for supporting distribution knowledge and a base for political decisions and future projects.

## General description

### Purpose

**Figure 1.**
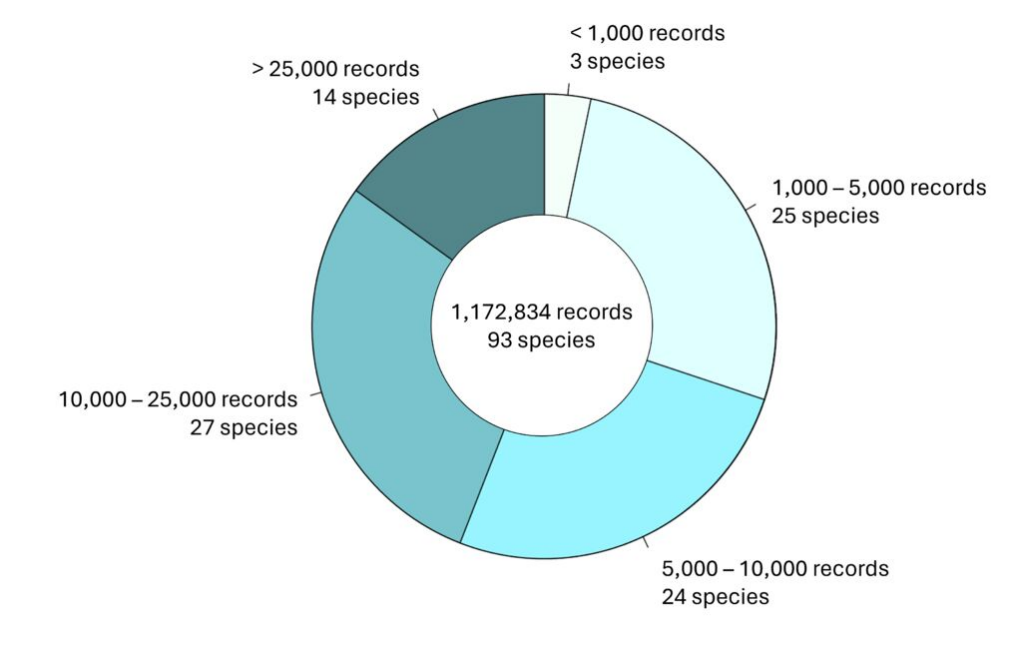
Number of georeferenced records per species listed in this dataset found in GBIF (gbif.org) as of July 2025. Most species exhibit less than 10,000 georeferenced observations worldwide. The R-Code to reproduce the figure is given in Suppl. material 2.

This data paper aims at making data available on carabid beetles in conventionally used agricultural landscapes. All specimens have been identified to species level along with detailed information on the date and location of each capture. This dataset includes some species for which only few published records are available in public international (gbif.org) databases (Fig. 1).

### Additional information

The background to the data collection was to answer questions about possible synergy effects between forest edges and flower strips in conventional agricultural landscapes. Two different seed mixtures were tested within the experimental framework following a split-plot design. Flower strips were implemented on half of all the study sites, while conventionally farmed fields with corn (*Zea mays*) for biogas production were chosen as control.

In the flower strips, two different seed mixtures were tested. One seed mixture corresponded to the mixture prescribed at federal state level for the creation of annual flower strips (at the time of the study) and consisted of seven non-native crop species, which were intended to provide flowering resources for honeybees (*Apis mellifera* Linnaeus, 1758) and which are commonly used as green manure. The second seed mixture was enriched with annual native herbs and consisted of 21 plant species in total.

## Project description

### Title

Mitigating the barrier effect of agricultural fields by means of semi-open network corridors

(Original title in German language: Minderung der Barrierewirkung von Agrarflächen mittels halboffener Verbundkorridore)

### Study area description

**Figure 2.**
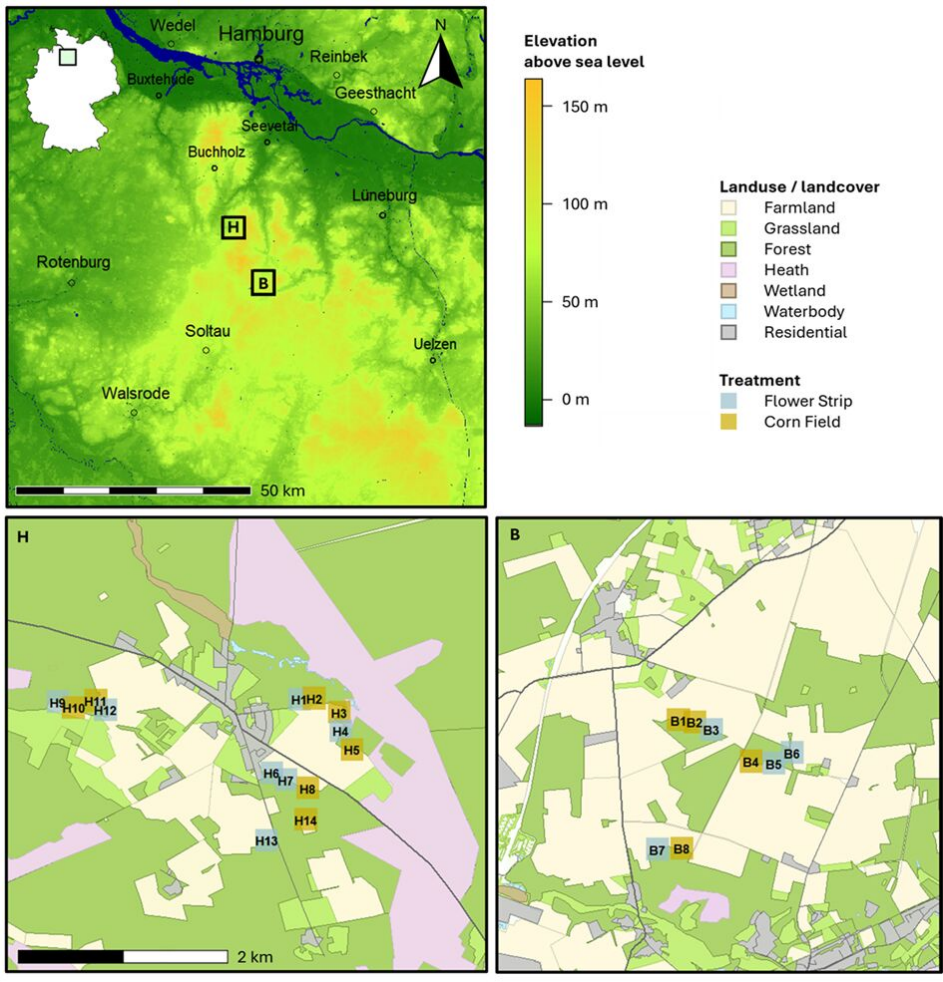
Locations of the study sites in the two regions H (= Harburg) and B (= Bispingen; Heidekreis) situated in North German Lowlands. **Top left**: Map showing the elevation profile of the surrounding landscape (SRTM; Farr and Kobrick (2000)). The small outline map of Germany with the marked square area in the top left-hand corner is intended to help assessing the location of the regions within Germany. **Bottom**: Detailed views of the regions depicting the exact location of the study sites and their spatial arrangement, as well as the surrounding landscape characteristics (OpenStreetMap). The map was created using a combination of the packages *maps* (Becker et al. 2024), *geodata* (Hijmans et al. 2024), *raster* (Hijmans 2024a), *terra* (Hijmans 2024b), *osmdata* (Padgham et al. 2017) and *prettymapr* (Dunnington 2024) within R version 4.2 (R Core Team 2024).

The study area is located in north-west Germany (Lower Saxony) (Fig. 2). The region represents a hilly landscape consisting of deposits of the penultimate glacial period (Saale glacial period) and is currently dominated by agricultural and forest areas with patches of heathlands. The latter are preserved as historical cultural landscapes for nature conservation purposes. The climate is temperate suboceanic (DWD 2025). Within this region, we selected 22 study sites. Fourteen of them are located in the District of Harburg and eight in the District of Heidekreis. The exact coordinates of the sites along with information on dominant tree species and stand age are listed in Suppl. material 1.

### Design description

The experiment followed a repeated partly split-plot design. We selected 22 fields where corn (*Zea mays*) was grown for the two years of the study with at least one edge adjacent to a forested area. On half of the fields (11), a flower strip 30 m wide and 200 m long was sown alongside the forest edge. Flower strips were divided into two equal parts, 100 m long and sown with two different seed mixtures. The first one is a standard seed mixture only containing seven non-native annual cultural flowering species commonly used for green manure. The second mixture was enriched with annual native herb species, thus totalling 21 plant species. Both flower strips were annual and sown each year in April. For details on seed mixture composition and implementation, see Grabener et al. (2021).

**Figure 3.**
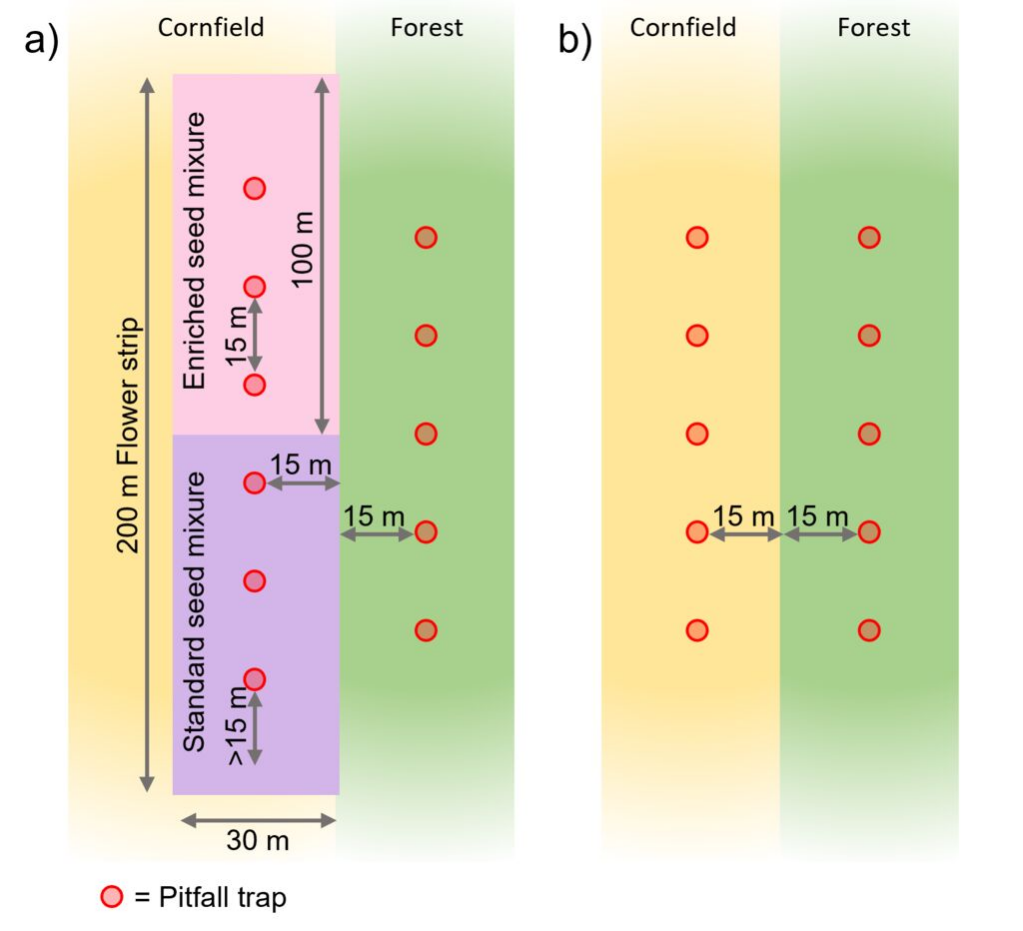
Experimental study design and spatial arrangement of the pitfall traps in the flower strips (**a**) and the corn field as well as in the adjacent forest (**b**).

Pitfall traps were installed at 15 m distance from the forest edge and from each other. Five traps were set in the forest as well as in the corn field treatment. In the flower strips, three traps per seed mixture were installed (Fig. 3). Traps were run continuously (except when the farmers worked the field), but for this dataset, only traps that were open between June to August were analysed, since the vegetation of the flower strips is well established within these months and effects of flower strips were expected to be most pronounced. If available, three traps per habitat, treatment and seed mixture (forest/corn field/flower strip standard/flower strip enriched) and time interval were evaluated, with any other traps obtained serving as a backup and not being evaluated. Where more than three traps were available, three traps were selected at random for evaluation. However, it was often the case that not all the traps set could be collected due to destruction, for example, by wild boar, birds or other unknown disturbances (Table 1).

### Funding

The project was funded by the German Federal Agency for Nature Conservation (BfN) with funds from the German Federal Ministry for the Environment, Nature Conservation and Nuclear Safety (BMU). Funding code: 35 16 820 300. This publication was funded by the Open Access Publication Fund of Leuphana University Lüneburg.

## Sampling methods

### Sampling description

Pitfall traps filled with so-called Renner-solution (40% ethanol, 30% aqua dest., 20% glycerine, 10% acetic acid, Renner (1980)) were installed in early spring (April 2017), except for the sites H13 and H14 which were installed one year later in April 2018. Traps were covered with wire mesh to prevent vertebrates from falling in. The sides of the wire were bent up so that even large specimens running on the ground could still fall in. Traps that went into the analysis were those that were collected within the months June to August of both years. The traps remained opened for 14 to 44 days, depending on weather conditions. Traps were changed more often when temperatures were high or after heavy rainfalls (to prevent deterioration due to dilution of the trapping solution). For each specimen, the following trap information was retrieved: site, treatment, habitat, (in case of flower strips) seed mixture, dates of opening and emptying of the trap and collector. Additionally, each trap was characterised by a unique ID code.

### Quality control

All collected individuals were identified by Swantje Grabener, Christin Juno Laschke, Sina Remmers and Lena Husemann in the laboratory and at least ten individuals per species (if available) were identified by the professional taxonomists Thorsten Assmann, Stephan Gürlich and Estève Boutaud. The taxonomy was checked to be compatible with the current species Red List for Germany (Schmidt et al. 2016).

### Step description

Traps were labelled in the field and further stored at 7°C until samples were processed and carabid beetles were identified in the laboratory. Data on species identity, number of individuals per species, the person who identified the species as well as the year of identification and, if necessary, the person who verified identification and the year of verification, were put into a table format together with the trap information. Specimens from the same trap were stored in vials containing Scheerpeltz-solution (70% ethanol, 5% acetic acid; Teichmann (1994)) for long-term storage. For each species, at least ten specimens (if available) were mounted and labelled with all trap and identification information.

The locations were retrieved using a standard GPS system. Coordinate uncertainty was set to 100 m as, for the coordinates, the centre of the study site was entered so that all traps from one study site had the same geographic coordinates thus ignoring the actual position of the traps. All formats follow GBIF Darwin Core specification to ensure interoperability with other international databases.

## Geographic coverage

### Description

The study was carried out in north German lowlands in the north-east of the Federal State of Lower Saxony. The majority of the study sites (14) were located in the Municipality of Undeloh in the District of Harburg and the remaining eight were located in the adjacent Municipality of Bispingen in the District of Heidekreis.

### Coordinates

53.1099 and 53.2300 Latitude; 10.0276 and 9.8936 Longitude.

## Taxonomic coverage

### Description

The dataset covers 93 species of the family Carabidae (including two species of the subfamily Cicindelinae) belonging to 30 genera (Tables 2, 3).

### Taxa included

**Table:** 

Rank	Scientific Name	Common Name
family	Carabidae	Ground beetles

## Temporal coverage

**Data range:** 2017-6-07 – 2017-10-01; 2018-6-18 – 2018-9-04.

### Notes

Traps that were open within the months June to August in the two years 2017 and 2018 were analysed and contributed to this dataset.

## Usage licence

### Usage licence

Creative Commons Public Domain Waiver (CC-Zero)

## Data resources

### Data package title

Ground beetle fauna of flower strips and forest edges in northern German lowlands’ conventional agricultural landscapes

### Resource link

https://ipt.pensoft.net/resource?r=car_agr_nw-ger

### Number of data sets

1

### Data set 1.

#### Data set name

occurrences

#### Data format

Darwin Core table (tab delimited)

#### Description

The dataset comprises counts of ground beetles (Coleoptera, Carabidae), which were recorded in agricultural landscapes using pitfall traps over a period of two years. Those traps from the summer months of June to August were evaluated. Traps were set in conventional corn fields (*Zea mays*), grown for biogas production, with and without flower strips sown with two different seed mixtures and in the neighbouring forest. The study sites represent typical agricultural landscapes in the northern German lowlands and the species composition is likely to be transferable to other predominantly agrarian Central European landscapes. The dataset comprises 34,413 individuals from 93 species. Most species are considered to be common at federal and state level. However, four species have been categorised as near threatened in Germany. At the level of the Federal State, four species are classified as endangered, nine species as vulnerable and one species as near threatened.

**Data set 1.** 

Column label	Column description
basisOfRecord	The specific nature of the data record (here: PreservedSpecimen).
occurrenceID	An identifier for the occurrence, possibly globally unique. Here it is constructed from a combination of parts of the host institution name, the site and the trap number, parts of the genus and the species names followed by a sequential number.
individualCount	The number of individuals present at the time of the occurrence.
organismQuantity	A number or enumeration value for the quantity of organisms. Here, an index of activity density (see organismQuantityType column).
organismQuantityType	The type of quantification system used for the quantity of organisms. Here, the activity density equals the individualCount per trap.
lifeStage	The age class or life stage of the organism(s) at the time the occurrence was recorded.
eventID	An identifier for the set of information associated with an event (something that occurs at a place and time). Here, it refers to the specific trap at a site (parentEventID).
parentEventID	An identifier for the broader event that groups this and potentially other events. Here, it refers to the site.
eventType	The nature of the event.
eventDate	The date-time during which an event was recorded. Here, it refers to the day on which the trap was set (first date entry) and emptied (second date after slash).
startDayOfYear	The earliest integer day of the year on which the event occurred. Here, it refers to the day when the trap was set.
endDayOfYear	The latest integer day of the year on which the event occurred. Here, it refers to the day when the trap was emptied.
year	The four-digit year in which the event occurred, according to the Common Era Calendar.
habitat	A category or description of the habitat in which the event occurred.
samplingProtocol	The names of, references to, or descriptions of the methods or protocols used during an event.
countryCode	The standard code for the country in which the location occurs.
stateProvince	The name of the next smaller administrative region than country (state, province, canton, department, region etc.) in which the location occurs.
county	The full, unabbreviated name of the next smaller administrative region than stateProvince (county, shire, department etc.) in which the location occurs.
municipality	The full, unabbreviated name of the next smaller administrative region than county (city, municipality etc.) in which the location occurs.
locality	The specific description of the place.
decimalLatitude	The geographic latitude (in decimal degrees) of the geographic centre of a location.
decimalLongitude	The geographic longitude (in decimal degrees) of the geographic centre of a location.
geodeticDatum	The ellipsoid, geodetic datum or spatial reference system (SRS), upon which the geographic coordinates given in decimalLatitude and decimalLongitude are based.
coordinateUncertaintyInMetres	The horizontal distance (in metres) from the given decimalLatitude and decimalLongitude describing the smallest circle containing the whole of the location.
identifiedBy	Person who assigned the taxon to the subject.
dateIdentified	The year on which the subject was determined as representing the taxon.
identificationVerificationStatus	A categorical indicator of the extent to which the taxonomic identification has been verified to be correct.
scientificName	The full scientific name, with authorship and date information.
acceptedNameUsage	The full name, with authorship and date information of the currently valid taxon.
kingdom	The full scientific name of the kingdom in which the species is classified.
phylum	The full scientific name of the phylum or division in which the species is classified.
class	The full scientific name of the class in which the species is classified.
order	The full scientific name of the order in which the species is classified.
family	The full scientific name of the family in which the species is classified.
genus	The full scientific name of the genus in which the species is classified.
specificEpithet	The name of the first or species epithet of the scientificName.
taxonRank	The taxonomic rank of the most specific name in the scientificName.

## Additional information

### General discussion on conservation implications

Within this study, there were 43 species that can be considered as abundant (> 25 individuals), making it possible to detect patterns of habitat preferences. Out of these species, two are considered threatened at the Federal or State level: (i) *Carabus convexus* is classified as *Vulnerable* in Lower Saxony and as *Near Threatened* in Germany. This species is wingless and eurytopic in the study region. Highest numbers of individuals were detected in the forest in both study regions (Harburg and Bispingen), highlighting the importance of forest fragments within the agricultural matrix for this species with limited dispersal power. This seems to be especially important in the light of a long-term decline in carabid beetle diversity in forests in the region (Homburg et al. 2019).

(ii) The herbivorous species *Harpalus froelichii* is classified as *Endangered* at the Federal State level. It was almost exclusively found within the flower strips sown with enriched seed mixture in the study region Bispingen. This species is known to prefer sunny dry bare soil (Rathmann and Piper 2007), the vegetation cover was lower in the flower strips sown with enriched seed mixture as native herbs grew less fast. Therefore, flower strips with enriched seed mixture exhibited more bare ground than those with standard seed mixtures.

**Figure 4.**
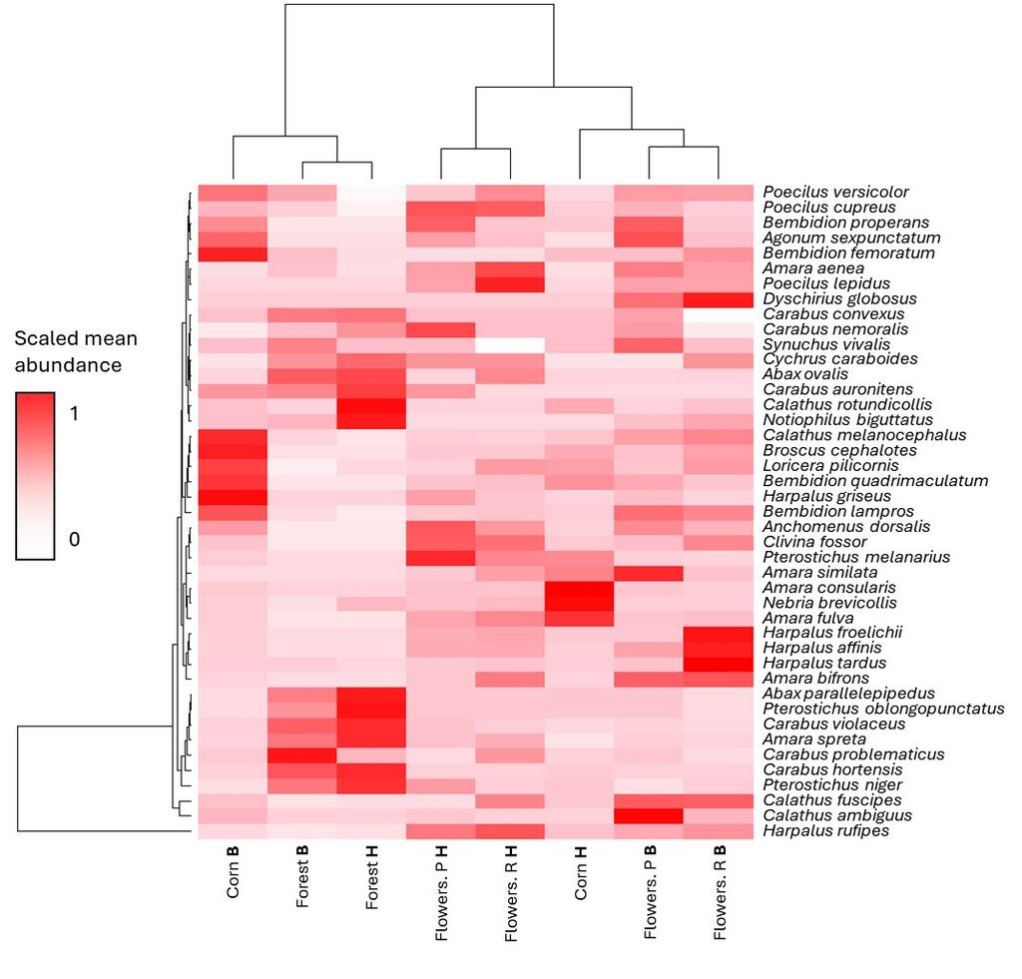
Similarity of mean species abundances for species with more than 25 individuals recorded in the dataset. Cornfield (= Corn), flower strips with standard species poor speed mixture (= Flowers. P) and with enriched seed mixture (= Flowers. R) and in forests (= Forest) in both study Regions Harburg (= H) and Bispingen (= B). The R-Code to reproduce the figure and to learn more about the underlying analysis is given in Suppl. material 2.

None of the abundant species occurred in only one habitat or treatment (Fig. 4). However, species with a preference for the field were found less frequently in the forest than forest species in the field. This is consistent with the results of other studies (Lacasella et al. 2014, Magura 2017). Forest sites had similar species assemblages throughout the study regions. In contrast, flower strips with different seed mixtures were similar within regions. In terms of species assemblages, corn fields differed from forests, flower strips and markedly from each other in the two regions, probably due to differences in management practices. In the Region Harburg, corn fields were undersown with buckwheat (*Fagopyrum esculentum* Moench, 1794) which could have made conditions more alike flower strips as undersowings have been shown to have variable effects on different carabid species (Vickermann 1978, Armstrong and McKinlay 1997).

Corn fields in the Region Bispingen had a specific ground beetle assemblage, especially characterised by a distinct group of mainly eurytopic predators including *Bembidion lampros*, *B. quadrimaculatum*, *Calathus melanocephalus* and *Loricera pilicornis* which were not found in high numbers in in the other district.

We would like to point out that, even within relatively small geographic scales, ground beetle assemblages differed markedly within similar treatments. This might have implications for the design of measures to promote biodiversity, especially insects that fulfil ecosystem services. These measures should be adapted to local conditions and present species assemblages and, thus, should be site specific.

### Phenological aspects and limitations

Not all traps placed at different sites were opened and emptied at the same day. This can potentially lead to some species being under- or over-represented due to the phenological aspects if the peak of activity of adult beetles is missed (e.g. Blubaugh et al. (2011), Castro et al. 2014), as well as due to temporal changes in spatiotemporal distribution of carabids (Knapp et al. 2019). These phenological aspects need to be considered when analysing the data for treatment effects.

The presented ground beetle dataset only focuses on captures from the summer months. However, differences in the ground beetle assemblage between the corn fields and the flower strips could be even more pronounced, especially in autumn after the harvest of the crop and in the winter if flower strip vegetation remains on the field.

## Supplementary Material

AA870236-9EEB-5452-A1AC-985886A6BC84 10.3897/BDJ.13.e161282.suppl1 Supplementary material 1 Study sites Data type Coordinates and characteristics of study sites Brief description Names of the study sites and their exact coordinates, field habitat and forest main tree species and average stand age (± 10 years). The given coordinated represent the centre of the study sites, which have an uncertainty of about 10 m. However, the sites themselves were larger (200 m length); thus, uncertainty for the position of the traps is set to 100 m. File: oo_1366173.csv https://binary.pensoft.net/file/1366173 Swantje Grabener, Thorsten Aßmann, Claudia Drees, Werner Haerdtle

4DCB9252-5B1E-501C-9E46-9FE39281CAF4 10.3897/BDJ.13.e161282.suppl2 Supplementary material 2 R-Script to reproduce the Figure 1 (GBIF-records) and Figure 2 (Heatmap and Dendrograms) Data type HTML document with R-Code Brief description Code to create the Fig. 1 and Fig. 4 of this publication. Additional R-packages need to be installed separately. Published data need to be downloaded from GBIF and is required to reproduce the figures. File: oo_1366935.html https://binary.pensoft.net/file/1366935 Swantje Grabener, Thorsten Assmann

## Figures and Tables

**Table 1.** Number of analysed trap samples per site, year and habitat.

**Site**	**Number of analysed samples 2017**		**Number of analysed samples 2018**	
	**Field**	**Forest**	**Field**	**Forest**
H1	8	5	11	3
H2	1	3	4	5
H3	2	3	-	-
H4	5	2	-	-
H5	-	3	-	-
H6	6	2	11	6
H7	13	3	10	4
H8	5	2	3	3
H9	12	6	11	8
H10	6	3	3	3
H11	12	3	12	5
H12	6	4	4	5
H13	-	-	12	6
H14	-	-	6	6
B1	6	4	6	5
B2	5	6	6	6
B3	16	6	12	6
B4	1	3	4	5
B5	7	5	10	6
B6	-	-	11	7
B7	10	3	17	9
B8	5	6	6	6
**Sum**	**126**	**72**	**159**	**104**

**Table 2.** Numbers of ground beetle species and specimens per genus.

**Genus**	**Number of species**	**Number of specimens**
* Abax *	2	498
* Agonum *	2	76
* Amara *	17	1,494
* Anchonemus *	1	132
* Anisodactylus *	1	12
* Asaphidion *	2	25
* Bembidion *	5	1,006
* Bradycellus *	2	2
* Broscus *	1	310
* Calathus *	6	709
* Calosoma *	1	7
* Carabus *	8	2,059
* Cicindela *	2	8
* Clivina *	1	142
* Cychrus *	1	28
* Dyschirius *	2	34
* Harpalus *	13	23,504
* Leistus *	2	14
* Loricera *	1	216
* Microlestes *	1	3
* Nebria *	2	116
* Notiophilus *	3	61
* Ophonus *	1	1
* Oxypselaphus *	1	13
* Poecilus *	3	233
* Pterostichus *	5	2,486
* Stomis *	1	4
* Syntomus *	2	5
* Synuchus *	1	26
* Trechus *	2	31
* Zabrus *	1	2

**Table 3.** Number of specimens per species and Red List Status (Red List Germany: Schmidt et al. (2016); Red List Lower Saxony: Assmann et al. (2002)). Nomenclature follows Schmidt et al. (2016). Abbreviations and Red List Status as in the IUCN Red Lists: LC: Low Concern, NT: Near Threatened, VU: Vulnerable, EN: Endangered.

**Species**	**German Red List Status**	**Lower Saxony Red List Status**	**Number of specimens**
*Abax ovalis* (Duftschmid, 1812)	LC	LC	27
*Abax parallelepipedus* (Piller & Mitterpacher, 1783)	LC	LC	471
*Agonum muelleri* (Herbst, 1784)	LC	LC	22
*Agonum sexpunctatum* (Linnaeus, 1758)	LC	LC	54
*Amara aenea* (De Geer, 1774)	LC	LC	34
*Amara apricaria* (Paykull, 1790)	LC	LC	8
*Amara aulica* (Panzer, 1796)	LC	LC	6
*Amara bifrons* (Gyllenhal, 1810)	LC	LC	290
*Amara brunnea* (Gyllenhal, 1810)	LC	LC	1
*Amara communis* (Panzer, 1797)	LC	LC	2
*Amara consularis* (Duftschmid, 1812)	LC	LC	193
*Amara convexior* Stephens, 1828	LC	LC	4
*Amara curta* Dejean, 1828	LC	VU	3
*Amara eurynota* (Panzer, 1796)	LC	VU	5
*Amara familiaris* (Duftschmid, 1812)	LC	LC	3
*Amara fulva* (O.F. Müller, 1776)	LC	LC	501
*Amara lunicollis* Schiödte, 1837	LC	LC	24
*Amara ovata* (Fabricius, 1792)	LC	LC	3
*Amara plebeja* (Gyllenhal, 1810)	LC	LC	2
*Amara similata* (Gyllenhal, 1810)	LC	LC	281
*Amara spreta* Dejean, 1831	LC	LC	134
*Anchomenus dorsalis* (Pontoppidan, 1763)	LC	LC	132
*Anisodactylus binotatus* (Fabricius, 1787)	LC	LC	12
*Asaphidion flavipes* (Linnaeus, 1760)	LC	LC	24
*Asaphidion pallipes* (Duftschmid,1812)	NT	VU	1
*Bembidion femoratum* Sturm, 1825	LC	LC	32
*Bembidion lampros* (Herbst, 1784)	LC	LC	585
*Bembidion properans* (Stephens,1828)	LC	LC	57
*Bembidion quadrimaculatum* (Linnaeus, 1760)	LC	LC	317
*Bembidion tetracolum* Say, 1823	LC	LC	15
*Bradycellus csikii* Laczó, 1912	LC	LC	1
*Bradycellus harpalinus* (Audinet-Serville, 1821)	LC	LC	1
*Broscus cephalotes* Linnaeus, 1758)	LC	LC	310
*Calathus ambiguus* (Paykull, 1790)	LC	LC	363
*Calathus cinctus* Motschulsky, 1850	LC	LC	8
*Calathus erratus* (C.R. Sahlberg, 1827)	LC	LC	10
*Calathus fuscipes* (Goeze, 1777)	LC	LC	1,066
*Calathus melanocephalus* (Linnaeus, 1758)	LC	LC	296
*Calathus rotundicollis* Dejean, 1828	LC	LC	32
*Calosoma auropunctatum* (Herbst, 1784)	NT	EN	7
*Carabus auronitens* Fabricius, 1792	LC	LC	53
*Carabus convexus* Fabricius, 1775	NT	VU	52
*Carabus glabratus* Paykull, 1790	LC	NT	10
*Carabus granulatus* Linnaeus, 1758	LC	LC	3
*Carabus hortensis* Linnaeus, 1758	LC	LC	937
*Carabus nemoralis* O.F. Müller, 1764	LC	LC	39
*Carabus problematicus* Herbst, 1786	LC	LC	627
*Carabus violaceus* Linnaeus, 1758	LC	LC	338
*Cicindela campestris* Linnaeus, 1758	LC	LC	1
*Cicindela hybrida* Linnaeus, 1758	LC	LC	7
*Clivina fossor* (Linnaeus, 1758)	LC	LC	142
*Cychrus caraboides* (Linnaeus, 1758)	LC	LC	28
*Dyschirius globosus* (Herbst, 1784)	LC	LC	29
*Dyschirius politus* (Dejean, 1825)	LC	VU	5
*Harpalus affinis* (Schrank, 1781)	LC	LC	127
*Harpalus calceatus* (Duftschmid, 1812)	LC	EN	9
*Harpalus distinguendus* (Duftschmid, 1812)	LC	LC	2
*Harpalus froelichii* Sturm, 1818	LC	EN	113
*Harpalus griseus* (Panzer, 1796)	LC	VU	30
*Harpalus latus* (Linnaeus, 1758)	LC	LC	4
*Harpalus luteicornis* (Duftschmid, 1812)	LC	EN	1
*Harpalus rubripes* (Duftschmid, 1812)	LC	LC	1
*Harpalus rufipalpis* Sturm, 1818	LC	LC	3
*Harpalus rufipes* (De Geer, 1774)	LC	LC	23,076
*Harpalus signaticornis* (Duftschmid, 1812)	LC	VU	10
*Harpalus smaragdinus* (Duftschmid, 1812)	LC	VU	15
*Harpalus tardus* (Panzer, 1796)	LC	LC	113
*Leistus ferrugineus* (Linnaeus, 1758)	LC	LC	4
*Leistus rufomarginatus* (Duftschmid, 1812)	LC	LC	10
*Loricera pilicornis* (Fabricius, 1775)	LC	LC	216
*Microlestes minutulus* (Goeze, 1777)	LC	LC	3
*Nebria brevicollis* (Fabricius, 1792)	LC	LC	108
*Nebria salina* Fairmaire & Laboulbène, 1854	LC	LC	8
*Notiophilus aquaticus* (Linnaeus, 1758)	LC	LC	2
*Notiophilus biguttatus* (Fabricius, 1779)	LC	LC	38
*Notiophilus palustris* (Duftschmid, 1812)	LC	LC	21
*Ophonus puncticeps* Stephens, 1828	LC	LC	1
*Oxypselaphus obscurus* (Herbst, 1784)	LC	LC	13
*Poecilus cupreus* (Linnaeus, 1758)	LC	LC	66
*Poecilus lepidus* (Leske, 1785)	LC	LC	39
*Poecilus versicolor* (Sturm, 1824)	LC	LC	128
*Pterostichus melanarius* (Illiger, 1798)	LC	LC	156
*Pterostichus niger* (Schaller, 1783)	LC	LC	1,965
*Pterostichus oblongopunctatus* (Fabricius, 1787)	LC	LC	356
*Pterostichus quadrifoveolatus* Letzner, 1852	NT	LC	7
*Pterostichus vernalis* (Panzer, 1796)	LC	LC	2
*Stomis pumicatus* (Panzer, 1796)	LC	LC	4
*Syntomus foveatus* (Geoffroy, 1785)	LC	LC	1
*Syntomus truncatellus* (Linnaeus, 1760)	LC	LC	4
*Synuchus vivalis* (Illiger, 1798)	LC	LC	26
*Trechus obtusus* Erichson, 1837	LC	LC	11
*Trechus quadristriatus* (Schrank, 1781)	LC	LC	20
*Zabrus tenebrioides* (Goeze, 1777)	LC	VU	2
