# Supplementary material for: Ground beetle fauna of flower strips and forest edges in northern German lowlands’ conventional agricultural landscapes (Coleoptera, Carabidae)
Source: Biodivers Data J. 2025 Aug 26;13:e161282. doi: 10.3897/BDJ.13.e161282 (PMC12405936; doi:10.3897/BDJ.13.e161282)
Supplement: Supplementary material 2 — R-Script to reproduce the Figure 1 (GBIF-records) and Figure 2 (Heatmap and Dendrograms) [file bdj-13-e161282-s002.html]

Supplement 2: R-Script


# Supplement 2: R-Script

#### Grabener et al., 2025 Biodiversity Data Journal

```
Occ <- read.csv("occurrence.csv")
```

# Figure 1

```
library(rgbif)

Species <- unique(paste(Occ$genus, Occ$specificEpithet))
GBIF <- data.frame(Species = Species, Count = NA)

for (sp in 1:length(Species)){
  Sp <- Species[sp]
  count <- occ_count(scientificName = Sp, hasCoordinate = TRUE)
  GBIF$Count[sp] <- count
}

GBIF$Kat <- 0
GBIF$Kat[GBIF$Count < 1000] <- 1
GBIF$Kat[GBIF$Count >= 1000 & GBIF$Count < 5000] <- 2
GBIF$Kat[GBIF$Count >= 5000 & GBIF$Count < 10000] <- 3
GBIF$Kat[GBIF$Count >= 10000 & GBIF$Count < 25000] <- 4
GBIF$Kat[GBIF$Count >= 25000] <- 5

pie(table(GBIF$Kat), labels = c("< 1 000", "1 000 - 5 000", "5 000 - 10 000",
                              "10 000 - 25 000", "> 25 000"),
    main = "number of observations",
    clockwise = TRUE, col = c("mintcream","lightcyan","cadetblue1", "cadetblue3", "cadetblue4"))
```

# Figure 4

```
library(reshape)
Occ2 <- Occ[c("county","habitat","eventID","scientificName","individualCount",
              "startDayOfYear", "endDayOfYear")]

mOcc <- melt(Occ2, id = c("county","habitat","eventID","scientificName", "startDayOfYear", "endDayOfYear"))
cOcc <- cast(mOcc, scientificName ~ variable, fun = "sum", value = "individualCount")
abundSpecies <- cOcc$scientificName[cOcc$individualCount >= 25]

DFabund <- subset(mOcc, scientificName %in% abundSpecies)

# standardise number to N per Day
mOcc$daysOpen <- mOcc$endDayOfYear - mOcc$startDayOfYear
mOcc$NperD <- mOcc$value / mOcc$daysOpen

cOcc2 <- cast(DFabund, scientificName ~ county + habitat, value = "NperD", fun = "mean")
dfOcc2 <- data.frame(cOcc2)[2:9]

names(dfOcc2) <- c("C.H","S.P.H","S.R.H","F.H","C.B","S.P.B.","S.R.B.", "F.B.")

m_abund <- as.matrix(dfOcc2)
m_abund[is.na(m_abund)] <- 0
rownames(m_abund) <- cOcc2$scientificName

Cols <- colorRampPalette(c("white","pink","red"))(100)
heatmap(m_abund, margins = c(6,12), scale = "row", col = Cols)
```
